# 高通量转录组测序技术研究过表达GPC5对A549细胞基因表达影响

**DOI:** 10.3779/j.issn.1009-3419.2016.08.11

**Published:** 2016-08-20

**Authors:** 海天 张, 国祥 王, 欣 杨, 满堂 邱, 林 许

**Affiliations:** 1 221004 徐州, 徐州医科大学临床医学系 Department of Clinical Medicine, Xuzhou Medical University, Xuzhou 221004, China; 2 221000 徐州, 徐州医科大学附属医院心胸外科 Department of Cardiothoracic Surgery, Affiliated Hospital of Xuzhou Medical University, Xuzhou 221000, China; 3 213003 常州, 常州市第一人民医院肿瘤内科 Department of Oncology, the First People's Hospital of Changzhou, Changzhou 213003, China; 4 210009 南京, 南京医科大学附属江苏省肿瘤医院胸外科/江苏省恶性肿瘤分子生物学及转化医学重点实验室 Department of Thoracic Surgery, Nanjing Medical University Affiliated Cancer Hospital, Jiangsu Key Laboratory of Molecular and Translational Cancer Research, Cancer Institute of Jiangsu Province, Nanjing 210009, China

**Keywords:** 磷脂酰肌醇蛋白聚糖-5, A549, 增殖, 基因表达, GPC5, A549, Proliferation, Gene expression

## Abstract

**背景与目的:**

磷脂酰肌醇蛋白聚糖-5(glypican-5, GPC5)是一个重要的抑癌基因, 然而GPC5对肺腺癌细胞增殖能力和基因表达的影响目前研究甚少。本研究拟在肺腺癌A549细胞中过表达GPC5以研究细胞增殖能力和基因表达变化情况。

**方法:**

通过慢病毒载体构建稳定过表达GPC5的A549细胞株, 通过Cell Counter Kit 8 (CCK8)、平板克隆和EdU实验检测细胞增殖能力; 通过高通量转录组测序研究基因表达变化。

**结果:**

相对于空白载体组, CCK8实验发现过表达GPC5可以明显抑制A549细胞的增殖速率; 平板克隆实验结果显示, 过表达GPC5之后A549细胞克隆形成能力下降(181±17 *vs* 278±23);EdU染色结果显示过表达GPC5后阳性染色细胞比例下降。转录组测序结果提示过表达GPC5之后, 2, 108个基因表达发生明显变化, 其中具有正性调节细胞增殖作用的基因明显下调。

**结论:**

过表达GPC5可以明显抑制肺腺癌细A549的增殖能力, 而且过表达GPC5后具有正性调节细胞增殖作用的基因表达下调。

## Abstract (en)

**Background and objective:**

Glypican-5 (GPC5) is an important tumor suppressor, while little is known about the impact of GPC5 on proliferation ability and gene expression in lung adenocarcinoma cell lines.Here, we stably overexpressed GPC5 in A549 cells and investigated the impact of cell proliferation ability and gene expression.

**Methods:**

A549 cells that stably overexpressed GPC5 were constructed by lentivirus.Cell counter kit 8 (CCK8), colony formation, EdU assay were conducted to analyze cell proliferation ability, and transcriptome sequencing was utilized to investigate gene expression profile.

**Results:**

CCK8 assay showed that compared with empty vector, overexpression of GPC5 significantly inhibited cell proliferation rate in A549 cells and the number of colony was also decreased (181±17 *vs* 278±23).EdU assay also confirmed the percentage of positive staining cells decreased after GPC5 overexpression.Transcriptome sequencing revealed that 2, 108 genes were differentially expressed after GPC5 overexpression.Among these differentially expressed genes, 47 genes of the Gene Ontology item "positive regulation of cell proliferation" were downregulated.

**Conclusion:**

Overexpression of GPC5 inhibited proliferation ability in lung adenocarcinoma A549 cells and genes with the function of "positive regulation of cell proliferation" were downregulated.

本课题组在前期研究中发现磷脂酰肌醇蛋白聚糖-5(glypican-5, GPC5)在非小细胞肺癌中扮演着抑癌基因的角色, 且GPC5在非小细胞肺癌组织中显著低表达, 而过表达GPC5可以显著抑制肺癌细胞的侵袭和迁移能力^[1]^。此后, 陆续有研究^[2, 3]^报道GPC5在肺癌中低表达且具有抑癌基因样作用。

目前的研究多认为GPC5是一个转移抑制因子, 而GPC5对细胞增殖影响的研究较少; 此外, GPC5的具体分子生物学机制尚未明确。因此, 在本研究中我们构建了稳定高表达GPC5的肺腺癌A549细胞株, 通过细胞生物学实验和高通量转录组测序手段来研究GPC5对肺腺癌细胞增殖能力和基因表达的影响

## 1 材料与方法

### 1.1 实验材料

人肺腺癌细胞株A549购于中国科学院上海细胞库; 慢病毒载体及稳定转染细胞株由上海吉凯生物公司完成; RNA提取试剂Trizol购于Invitrogen公司。1640培养基和胎牛血清购于Gibco公司, Cell Counter Kit8(CCK8)和EdU试剂盒购于南京凯基生物公司。

### 1.2 细胞培养

肺腺癌细胞株A549在含10%FBS的1640培养液中, 37 ℃、5%CO_2_保持饱和湿度培养, 2天-3天传代一次^[4]^。

### 1.3 CCK8实验

将处于对数生长期的细胞接种于96孔板, 每孔接种3, 000个细胞, 每组设置5个复孔; 分别在接种细胞贴壁后的0 h、24 h、48 h、72 h、96 h吸去培养基, 每孔加入100 μL培养液和10 μL CCK8试剂, 孵育2 h后使用自动酶标仪检测450 nm波长处吸光值^[5]^。

### 1.4 平板克隆实验

将处于对数生长期的细胞接种于6孔板, 每孔接种200个细胞, 每4天换液一次。2周后吸去培养基, 使用甲醇将细胞克隆固定, 然后用结晶紫染液染色, 计数每个孔中克隆形成数目并拍照。

### 1.5 EdU实验

将处于对数生长期的细胞均匀接种与盖玻片上, 待细胞贴壁后使用凯基EdU试剂盒进行染色固定并拍照。

### 1.6 转录组测序

GPC5稳定转染和空白对照的A549细胞由Trizol法提取RNA, 转录组测序及数据分析由上海烈冰生物有限公司完成。

### 1.7 统计学方法

所用统计分析使用SPSS 18.0统计软件完成。两个样本均数比较采用独立样本*t*检验, *P* < 0.05为差异有统计学意义。

## 2 结果

### 2.1 过表达GPC5抑制A549细胞增殖速率和克隆形成能力

相对于空白载体组, CCK8结果提示稳定过表达GPC5后, A549细胞的增殖速率被显著抑制, 且以接种后第三天后抑制效果最明显(图 1A)。在平板克隆形成实验中, 过表达GPC5后A549细胞形成的克隆数目显著少于空白载体组(181±17 *vs* 278±23, 图 1B), 且具有统计学差异。

**1:**
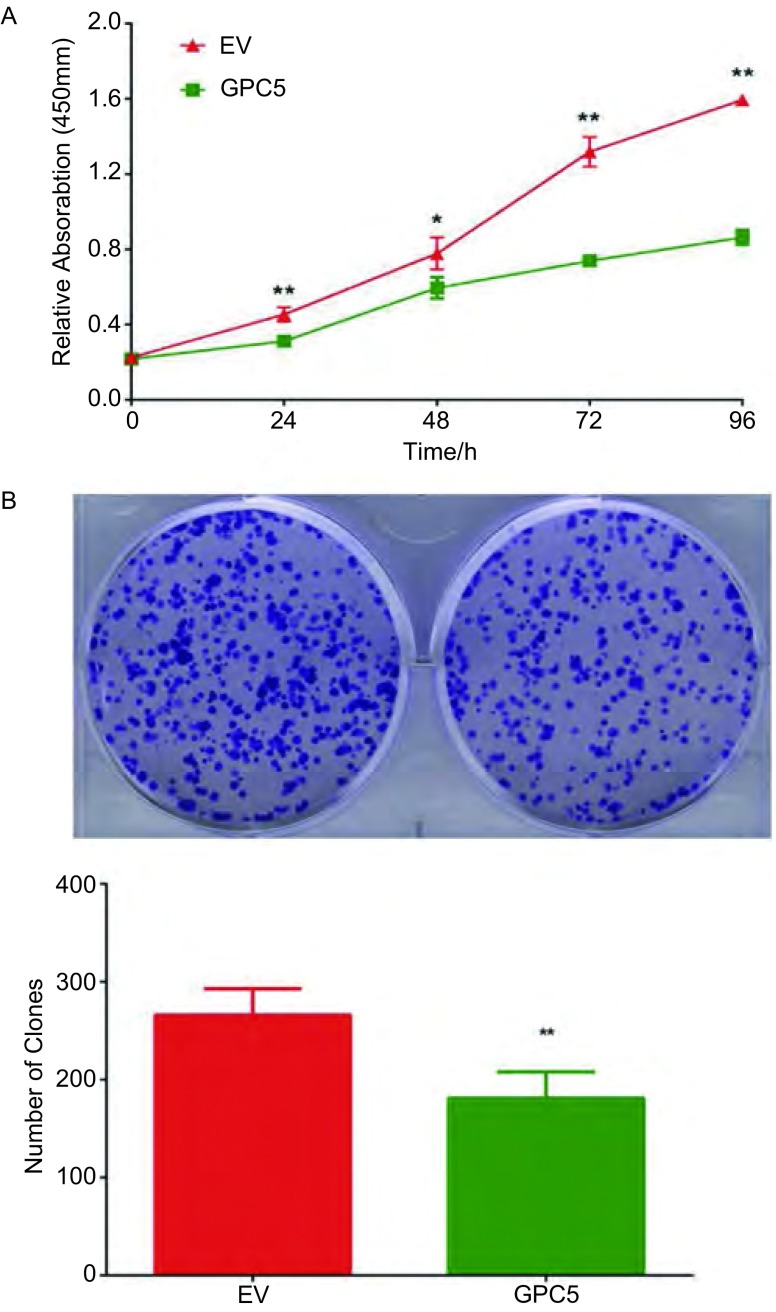
相对于空白载体组, 过表达GPC5显著抑制了A549细胞的增殖速率(A), 克隆形成数目也显著减少(181±17 *vs* 278±23)(B)。EV:空载体组; GPC5:过表达GPC5组; ^*^P < 0.05, ^**^P < 0.01。 Compared with empty vector, overexpression of GPC5 significantly inhibited cell proliferation rate (A) and colony formation number (B).EV:empty vector; GPC5:overexpression of GPC5;^*^P < 0.05, ^**^P < 0.01.

### 2.2 过表达GPC5抑制A549细胞增殖能力

如图 2所示, 稳定转染GPC5之后, EdU染色阳性的细胞比例显著低于空白载体组。CCK8、平板克隆形成和EdU结果证实:过表达GPC5可以抑制肺腺癌细胞A549的增殖能力。

**2:**
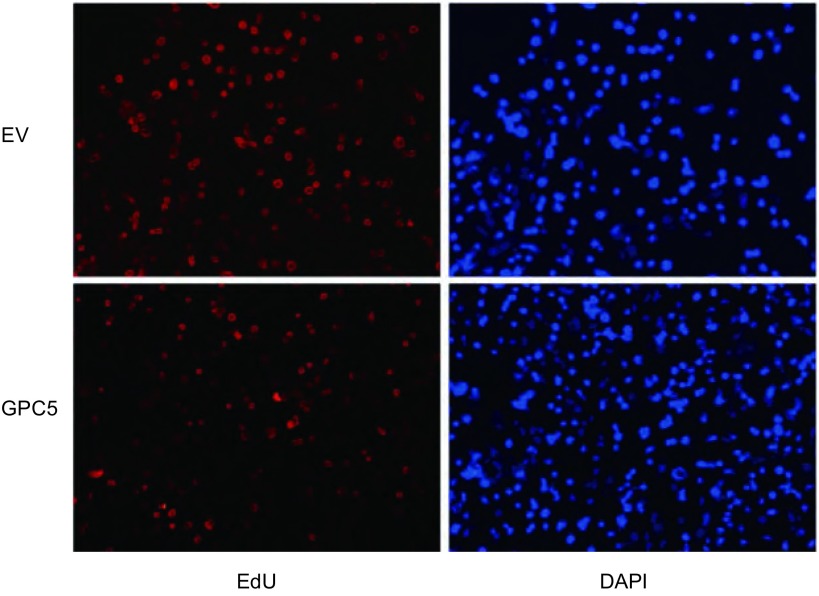
相对于空白载体组, 过表达GPC5组阳性细胞比例显著降低。EV:空载体组; GPC5:过表达GPC5组。 Compared with empty vector, overexpression of GPC5 decreased the percentage of positive staining cells.EV:empty vector; GPC5:overexpression of GPC5.

### 2.3 过表达GPC5抑制增殖相关基因表达

为了研究过表达GPC5对A549细胞基因表达变化影响, 我们将稳定转染GPC5和空白载体组的A549进行了高通量转录组测序。转录组测序结果显示, 稳定转染GPC5之后有876个基因表达显著升高, 而1, 232个基因表达显著降低。对下调基因进行基因本体论(gene ontology, GO)分析发现具有“positive regulation of cell proliferation”功能的47个基因被显著富集(图 3和表 1)。转录组测序和GO分析结果提示过表达GPC5可以下调具有正性调节细胞增殖功能基因的表达, 进而抑制细胞增殖能力。

**3:**
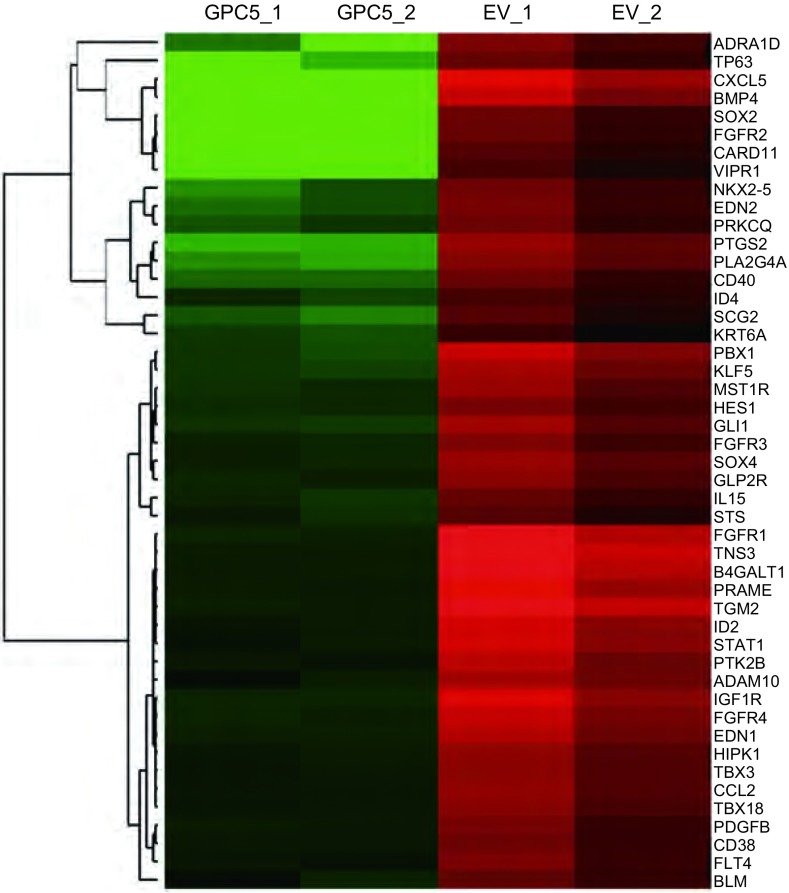
过表达GPC5后, 47个具有正性调节细胞增殖功能的基因显著下调。EV:空载体组; GPC5:过表达GPC5组; 绿色:下调基因; 红色:上调基因。 Compared with empty vector, 47 genes of Gene Ontology item "positive regulation of cell proliferation" were downregulated.EV:empty vector, GPC5:overexpression of GPC5;green:downregulated genes; red:upregulated genes.

**1:** 47个具有正性调节细胞增殖功能的基因 47 downregulated genes of Gene Ontology item "positive regulation of cell proliferation"

Symbol	Description	Log2FC
*CXCL5*	C-X-C Motif Chemokine Ligand 5	-20
*BMP4*	Bone Morphogenetic Protein 4	-20
*PBX1*	Pre B Cell Leukemia Homeobox 1	-3.163, 6
*PTGS2*	Prostaglandin-Endoperoxidase Synthase 2	-6.575, 86
*FGFR1*	Fibroblast Growth Factor Receptor 1	-1.935, 44
*ADRA1D*	Adrenoceptor Alpha 1D	-6.038, 9
*IGF1R*	Insulin Like Growth Factor 1 Receptor	-2.022, 54
*SOX2*	SRY (Sex Determining Region Y)-Box 2	-20
*TNS3*	Tensin-3	-1.551, 45
*B4GALT1*	Beta-1, 4-Galactosyltransferase 1	-1.544, 37
*FGFR2*	Fibroblast Growth Factor Receptor 2	-20
*FGFR4*	Fibroblast Growth Factor Receptor 4	-1.854, 04
*TP63*	Tumor Protein P63	-7.436, 53
*MST1R*	Macrophage Stimulating 1 Receptor	-2.411, 87
*EDN2*	Endothelin-2	-4.065, 38
*EDN1*	Endothelin-1	-1.76919
*NKX2-5*	NK2 Homeobox 5	-4.213, 1
*KLF5*	Kruppel Like Factor 5	-3.0153, 7
*CD40*	Tumor Necrosis Factor Receptor Superfamily Member 5	-4.545, 17
*PRAME*	Preferentially Expressed Antigen In Melanoma	-1.452, 48
*SOX4*	SRY (Sex Determining Region Y)-Box 4	-1.910, 7
*GLI1*	GLI Family Zinc Finger 1	-2.793, 53
*CARD11*	Caspase Recruitment Domain Family Member 11	-20
*ID2*	Inhibitor Of DNA Binding 2, HLH Protein	-1.227, 36
*GLP2R*	Glucagon-Like Peptide 2 Receptor	-1.895, 55
*STAT1*	Signal Transducer And Activator Of Transcription 1	-1.120, 7
*HES1*	Homeodomain Interacting Protein Kinase 1	-2.246, 06
*FGFR3*	Fibroblast Growth Factor Receptor 3	-1.928, 27
*HIPK1*	Homeodomain Interacting Protein Kinase 1	-1.363, 57
*PLA2G4A*	Phospholipase A2 Group IVA	-5.990, 5
*SCG2*	Secretogranin Ⅱ	-4.430, 16
*TGM2*	Transglutaminase 2	-1.645, 2
*VIPR1*	Vasoactive Intestinal Peptide Receptor 1	-20
*PRKCQ*	Protein Kinase C Theta Type	-3.251, 94
*IL15*	Interleukin-15	-2.350, 61
*CCL2*	Chemokine (C-C Motif) Ligand 2, Isoform CRA_A	-1.180, 96
*TBX3*	T-Box 3 (Ulnar Mammary Syndrome), Isoform CRA_C	-1.184, 44
*PTK2B*	Protein-Tyrosine Kinase 2-Beta	-1.106, 44
*TBX18*	T-Box Transcription Factor TBX18	-1.138, 41
*PDGFB*	Platelet Derived Growth Factor Subunit B	-1.424, 03
*CD38*	CD38 Molecule	-1.244, 04
*STS*	Steroid Sulfatase (Microsomal), Isozyme S	-1.773, 26
*FLT4*	Fms-Related Tyrosine Kinase 4	-1.011, 82
*ID4*	Inhibitor Of DNA Binding 4, HLH Protein	-2.818, 76
*ADAM10*	ADAM Metallopeptidase Domain 10, Isoform CRA_B	-1.085, 67
*KRT6A*	Keratin 6A	-3.236, 74
*BLM*	Bloom Syndrome Recq Like Helicase	-1.163, 52
FC:fold change.		

## 3 讨论

GPC5是一种细胞表面硫酸乙酰肝素蛋白多糖, GPC5可以通过糖基-磷脂酰肌醇锚定在细胞膜表面。*GPC5*基因属于磷脂酰肌醇蛋白聚糖(heparan sulphate proteoglycans, HSPGs)家族, 该家族有6个成员, 分别为GPC1到GPC6^[6, 7]^。HSPGs家族成员与多种肿瘤发生进展相关, 如GPC3在肝癌中显著高表达^[8]^, 而GPC1可以抑制胰腺癌细胞增殖^[9]^。而目前对于GPC5与肿瘤的报道相对较少, 对于GPC5发挥抑癌作用的分子生物学机制尚不清楚。

本课题组已报道在肺癌细胞中, 过表达GPC5可以显著抑制SK-MES1细胞的侵袭、迁移能力, 在本研究中我们进一步在肺腺癌细胞A549中探讨GPC5对细胞增殖能力和基因表达的影响。通过CCK8、平板克隆和EdU实验, 我们证实了过表达GPC5可以显著抑制肺腺癌A549细胞的增殖能力。进一步对细胞进行高通量转录组测序发现, 相对于空白载体组, 过表达GPC5后, 2, 108个基因的表达发生显著变化。进一步分析发现过表达GPC5之后, 具有正性调节细胞增殖的基因显著下调, 例如CXCL5^[12]^、SOX4^[11, 12]^。作为一个细胞膜蛋白, Li等^[6]^曾推测GPC5可能通过刺激或者抑制下游的信号转导通路来调控基因表达, 如通过Wnt、hedgehog和FGF信号通路。本研究发现过表达GPC5可以导致众多基因表达发生显著变化, 而其中的具体分子生物学机制还需进一步研究。

本研究结果证实过表达GPC5可以显著抑制肺腺癌细胞的增殖能力, 而且过表达GPC5后具有正性调节细胞增殖作用的基因表达下调, 具体的信号通路机制还需进一步实验验证。
